# Common peroneal nerve injury after tibial plateau fractures: A case series^☆^^☆☆^

**DOI:** 10.1016/j.tcr.2023.100916

**Published:** 2023-08-21

**Authors:** Jaime Garcia-Fernandez, Alexa Belcheva, Will Oliver, John F. Keating

**Affiliations:** a The University of Edinburgh Medical School, 47 Little France Crescent, EH16 4TJ Edinburgh, Scotland, UK; b The Royal Infirmary of Edinburgh, Trauma and Orthopaedics Surgery, 51 Little France Crescent, Old Dalkeith Road, EH16 4SA Edinburgh, Scotland, UK

**Keywords:** Tibial plateau fracture, Common peroneal nerve palsy, Foot drop, Trauma, Complications, Prognosis

## Abstract

**Introduction:**

Common peroneal nerve (CPN) injury is a rare but significant complication of knee trauma. Given its low incidence, there is limited published evidence, but reports have shown dislocations and fractures associated with varus deformity are more likely to injure the nerve, causing foot drop. This study aims to document the incidence and outcome of CPN palsy in tibial plateau fractures (TPF).

**Methods:**

We reviewed 746 cases of tibial plateau fractures treated between 2011 and 2020. We analysed patients' demographics, injury mechanisms, clinical course, and complications, and identified those with CPN palsies. Fractures were classified using the Schatzker, Luo and AO/OTA systems. The details of the CPN injury, including nerve conduction studies, duration of symptoms and outcome were recorded.

**Results:**

We identified 11 patients who had concurrent TPFs and CPN palsies, an overall incidence of 1.47 %. Most fractures involved the medial column (n = 9), with the C3 fragmentary TPF pattern being the most common (n = 4). The incidence of CPN injury was higher in medial fractures (5 %) and bicondylar fractures (3 %). We also found that most patients (n = 9) recovered full neurological function within 2 years.

**Discussion:**

This is the first study looking at a patient cohort sustaining concurrent TPFs and CPN injuries. It is a rare complication but should be looked for in high-risk medial and bicondylar fractures. We found that prognosis is better in TPF-associated CPN palsy than in other knee trauma, and that the majority of patients can expect a full recovery of nerve function.

## Highlights

• Common peroneal nerve palsy is a rare but significant complication of tibial plateau fractures.
• CPN palsy is more likely to happen in medial plateau fractures causing varus stress, bicondylar, and multifragmentary TPFs.
• CPN palsy has better outcomes after TPFs than other knee injuries (dislocation), with most patients recovering nerve function.

## Introduction

Common peroneal nerve (CPN) injury is a known complication of injuries, fractures, and lesions at the lower limb, most commonly associated with knee injuries [1]. Complete CPN palsy results in a foot drop, causing loss of dorsal foot sensation and inability to dorsiflex and evert the ankle and hindfoot [2]. Several mechanisms of injury have been identified, and they are usually classified as traumatic (including fractures, dislocations, and direct impact) or atraumatic (iatrogenic, external compression, nerve tumours, etc.) [3].

CPN injury is the most common mononeuropathy in the lower limb and the third most common focal neuropathy in the body, after carpal tunnel syndrome and ulnar neuropathies [4,5]. It is most commonly caused by traumatic aetiologies, especially in knee dislocations, where is has been found to occur in 16–40 % of cases [6]. In terms of injury mechanism, it is usually deforming forces causing varus that are associated with injury to the nerve since the nerve is tethered to the neck of fibula and prone to traction injury with this deforming force [7,8]. In the published literature we have found only one previously published case report of a CPN injury associated with a lateral tibial plateau fracture [8].

The incidence of CPN injury in fractures is poorly studied, and there is some debate as to which specific fracture patterns increase the risk of it. Previous research has shown that diaphyseal fractures of the tibia are the most common associated with CPN injury, followed by distal and proximal tibial fractures [9]. These two latter ones were found to happen at similar rates but at a much lower incidence [9]. Given their low incidence, previous studies of CPN injury in proximal and distal TPFs have had small sample sizes and therefore limited evidence, but a previous review of CPN injury associated with knee trauma has estimated the risk to be 1 % of tibial plateau fractures [10].

However, no previous studies have specifically evaluated the risk of CPN palsy with tibial plateau fractures [11,13]. There is reason to consider that fractures associated with varus forces at the time of injury may be associated with the risk of CPN traction injury, but no published studies have documented the incidence of this. There is also lack of evidence to link which type of plateau fracture is more likely to be associated with the risk of CPN injury.

The aim of the present study was to evaluate the incidence of CPN palsy in tibial plateau fractures (TPFs), document the fracture patterns most commonly associated with this nerve injury and determine the prognosis.

## Materials and methods

This study is a sub-study of a larger project looking at general epidemiology of tibial plateau fractures over a 10-year period treated at the single centre for orthopaedic surgery in an urban Scottish population. For this descriptive, retrospective study, we first identified all the patients who had suffered a tibial plateau fracture between the 1st of January 2011 and the 31st of December 2020. We did a hospital-system database search for any patients with a clinical note entry containing the words “tibial”, “plateau” and “fracture” in that time period.

We then reviewed their clinical and radiographic records to exclude any incorrectly classified injuries. We also excluded patients with extra-articular proximal tibial fractures, isolated tibial spine fractures, other knee injuries or other degenerative conditions. This yielded 761 patients. Of this group, 15 patients were lost to follow-up and removed from the study, which left a final cohort of 746 patients sustaining TPFs over the time period of the study.

For these patients, we reviewed their demographics in detail, and recorded patients' age, sex, deprivation index, occupation, past medical history, smoking status, alcohol intake and body mass index (BMI).

We then classified these fractures according to the Schatzker, Luo and AO [14,15,17] classifications after reviewing their radiographs and CT scans. We also recorded concomitant fibular head fractures and other clinical parameters such as side of injury, mechanism of injury, other coexisting injuries and if it was an open fracture.

Finally, we noted the details of how the fractures were managed (i.e. surgically or not, what kind of surgery or joint support, etc.). We noted patients' clinical course, how long they were followed up for at the orthopaedics department, any need for further surgery (e.g. knee joint replacements or removal of metalwork) and any other early or long-term complications. For the purposes of the present study, we identified any patients with CPN palsy, and documented the epidemiology of this subset of patients and the outcomes. Here, we highlighted the patients where common peroneal nerve palsy or foot drop had been clinically diagnosed, and analysed them individually as a sub-study of the previous epidemiological study.

In terms of management of the fractures, patients were managed operatively with open reduction and internal fixation (ORIF) or non-operatively with a knee cast or Donjoy brace as appropriate for each fracture. ORIF was performed with anatomic specific plateau fixation plates, augmented with calcium phosphate cement in selected cases.

Rehabilitation protocol was standard for tibial plateau fixation as per our Trust guidelines: non-weightbearing for 6–8 weeks and then partial weightbearing progressing to full weightbearing by weeks 10–12 post-injury. In cases where patients had a CPN injury, they were provided an ankle foot orthosis until nerve function returned. All patients received physiotherapy with the aim of restoring joint range of motion to full knee extension and beyond 90 degrees of flexion by 8 weeks post-injury. In patients with CPN injury, physiotherapy was focussed on encouraging active dorsiflexion strengthening of the tibialis anterior muscle to prevent atrophy and speed up recovery.

Patients with incomplete resolution of symptoms underwent nerve conduction studies, and nerve grafting was performed in selected cases with advice from neurology specialists. Neuropathic pain caused by common peroneal nerve injury was managed with either gabapentin, amitriptyline, pregabalin or duloxetine as per our Trust guidelines. The choice of medication was decided on a case-by-case basis depending on patient comorbidities and other medications. These patients were also referred to the pain clinic service at the Royal Infirmary of Edinburgh if the pain was difficult to control.

All statistical analysis was done using SPSS 24. Continuous data was tested for normality using Shapiro-Wilks tests. Normally distributed data was summarised using the mean of the sample ± standard deviation (SD) and compared using unpaired samples *t*-tests. Non- parametric data was summarised using the median and inter-quartile range and compared using Mann-Whitney *U* tests. Continuous data was compared using Pearson's correlation coefficient and, Chi-squared tests were used for categorical data. A p-value of <0.05 was considered statistically significant.

## Results

The study cohort comprised 746 patients who sustained tibial plateau fractures over the time period 2011–2020, and 11 of them (1.47 %) were found to have a concomitant common peroneal nerve injury. Patient details are summarised in Table 1. One patient was diagnosed post-operatively and had no signs of foot drop before surgery; all other patients were diagnosed at the time of presentation to A&E. There were 8 male and 3 female patients with an average age of 44 years (range 28 to 66 years). The mean age was not significantly different from the main cohort of all patients sustaining TPFs who had an average age of 52.77 ± 18.61 (p = 0.11).

**Table 1:** Patient details summary.

Patient ID	Age	Gender	AO	Schatzker	Luo	Treatment	Outcome of CPN	Other knee injuries
1	54	Female	B1.2	4	Medial	External fixation	No recovery	Knee dislocation, medial femoral condyle fracture
2	34	Male	B1.3	4	Medial	Knee cast	Partial recovery	None
3	28	Male	B2.2	3	Medial	Hinged knee brace	No recovery	Lateral collateral ligament rupture, knee dislocation
4	55	Male	B3.2	4	Medial	ORIF	Resolved	Fibular head fracture
5	39	Male	B3.1	2	Lateral	ORIF	Resolved	Medial collateral ligament sprain
6	66	Female	B2.2	3	Zero	Hinged knee brace	Resolved	Patellar fracture
7	53	Male	C3.1	5	Medial-lateral-posterior	ORIF	Resolved	Fibular head fracture
8	30	Male	B1.3	1	Medial	ORIF	Resolved	None
9	39	Male	C3.3	6	Medial-lateral-posterior	ORIF	Resolved	Proximal fibular head fracture
10	53	Male	C3.1	5	Medial-lateral-posterior	ORIF	Resolved	Fibular head fracture
11	30	Female	C3.3	5	Medial-lateral-posterior	ORIF	Resolved	None

Men were more likely to sustain these types of injuries (n = 8, 73 %), and accounted for a significantly higher proportion than in the main group of TPFs (n = 326, 45 %, p = 0.04). All fractures were closed fractures, and both legs were similarly affected, with five patients having right sided injuries, and 6, left sided ones. Patient's BMIs were normally distributed (p = 0.52) with an average of 25.71 ± 2.43 kg/m^2^, which was similar to the main group of patients (27.57 ± 7.94, p = 0.44).

The mechanisms of injury of these fractures were variable, with a fall from standing (FFS) being the most common (n = 4, 36 %), followed by road traffic accidents (RTA, n = 3, 28 %). This was similar to the main group of patients sustaining TPFs, where FFS was the most common mechanism of injury (n = 221, 29.6 %), and any differences were not significant. Around half of these patients (n = 6, 56 %) had a concomitant fibular head injury, which is significantly more than the proportion of these fractures happening in the main group (n = 123, 16.5 %, p < 0.01). In addition, the same proportion of patients had some other kind of injury to the knee (including soft tissue injury, knee dislocation and fibular head, patellar or femoral condyle fractures, as detailed in Table 1). This meant that the total proportion of isolated TPF with no other knee injury of 18 % (n = 2), which was significantly lower than in the main group (n = 457, 61.3 %, p = 0.01).

The majority of these patients (n = 8, 73 %) needed surgery to fix their TPFs, with most of them (n = 7, 88 % of the operated cohort) undergoing open reduction and internal fixation with plating. One was treated with external fixation as the definitive method of management. Most of these surgeries were uncomplicated, but one patient was clinically anaemic and needed two units of blood transfused, another had a superficial wound infection needing oral antibiotics, and a third patient suffered from both of these post-surgical complications. The other 3 patients were all managed with hinged knee braces and had an uneventful rehabilitation with physiotherapy.

Most fractures showed varus deformity on initial radiographic assessment (n = 10, 91 %), with the only fracture not showing this being the lateral tibial plateau fracture where the CPN palsy was only first mentioned in post-operative notes, indicating this was likely to have been an iatrogenic nerve injury.

When looking at the fracture classification, B-type fractures were more common, with 6 patients (56 %) being classified in this group. However, in the main group, there were 624 B-type fractures and 122 C-types. Therefore, 4.09 % of patients with C-type fractures had CPN injury, compared to only 0.96 % of patients with B-type fractures, making CPN palsy four times more common in C-type fractures (p = 0.01). More specifically, the most common fracture pattern identified was the C3 pattern (n = 4, 36 %), which was significantly more common than in the main group (n = 78, 10.5 %, p = 0.02).

**Fig. 1:**
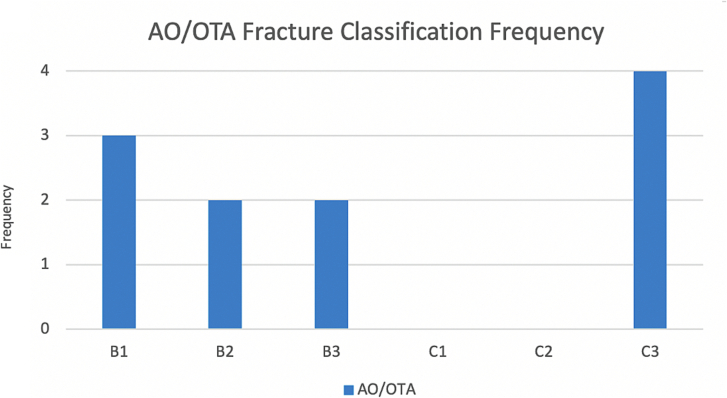
Frequency graph based on AO/OTA classification of TPFs with CPN injury.

The spread between the different specific types of AO/OTA classification was balanced, with three patients having split fractures (B1, 27 %), one patient with a depression fracture (B2, 9 %), two with split depression fractures (18 %) and another with a simple metaphyseal fracture (9 %, B3; Fig. 1). The remaining four patients had type C3 multifragmentary bicondylar plateau fractures (C3, 36 %). When assessing laterality of B-type fractures according to the AO/OTA classification, one patient had a lateral plateau fracture (B3.1, 17 %), whilst 5 patients had medial TPFs (B1.2, B1.3, B2.2 or B3.2; 83 %).

B and C-type fractures had similar lengths of follow-up in the CPN subgroup (34.83 ± 26.47 weeks in B-type vs 24.17 ± 9.47 weeks in C-type, p = 0.41). Interestingly, these were significantly different in the main TPF group, with patients B-type fractures being discharged from orthopaedics earlier (20.39 ± 20.36 vs 36.19 ± 42.22 weeks, p < 0.01). This difference could be explained by the fact that B-type fractures with associated CPN injury had a significantly longer follow-up time than B-type fractures without nerve injury (p = 0.04).

**Fig. 2:**
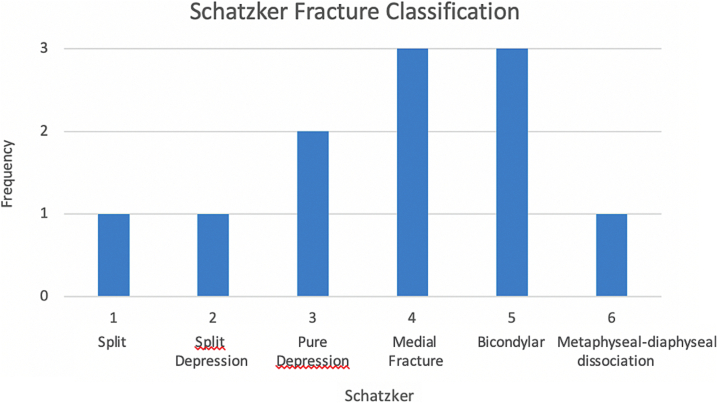
Frequency graph based on Schatzker classification of TPFs with CPN injury.

The most common Schatzker classifications were patterns IV and V (n = 3, 27 %), followed by pattern III (n = 2, 18 %), and all other patterns only had one patient (Fig. 2). When using this classification to compare unicondylar and bicondylar fractures, unicondylar fractures were more common (patterns I, II, III and IV, n = 7, 64 %), as in the main TPF group (n = 612, 82 %). However, bicondylar fractures (patterns V and VI) happen in a higher proportion in the CPN group (n = 4, 36 %) than in the main TPF group (n = 134, 18 %), giving a higher incidence of CPN in bicondylar fractures (2.99 %) than in unicondylar fractures (1.14 %).

Finally, when considering the Luo classification, the majority of fractures involved the medial column of the plateau (n = 9, 82 %). Four of these fractures involved all three columns (36 % of the total group), and the other five just involved the medial column, and only one patient had an only lateral column fracture. These proportions are significantly different (p < 0.01) to the main group of patients with tibial plateau fractures, where 111 (15 %) patients had fractures of just the medial column, 267 (36 %) involved the lateral column only, and 74 (9.9 %) involved all three. In total, 196 fractures (26 %) of these fractures involved the medial column of the tibial plateau, giving an incidence of CPN injury in these fractures of 4.59 %.

**Fig. 3:**
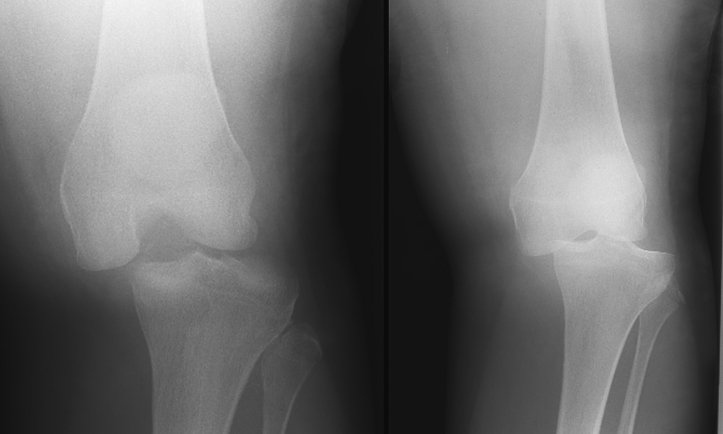
AP radiographs of both patients with tibial plateau fractures with complete dislocation and total loss of joint congruity. These fractures are anteromedial and therefore harder to visualize on AP views.

**Fig. 4:**
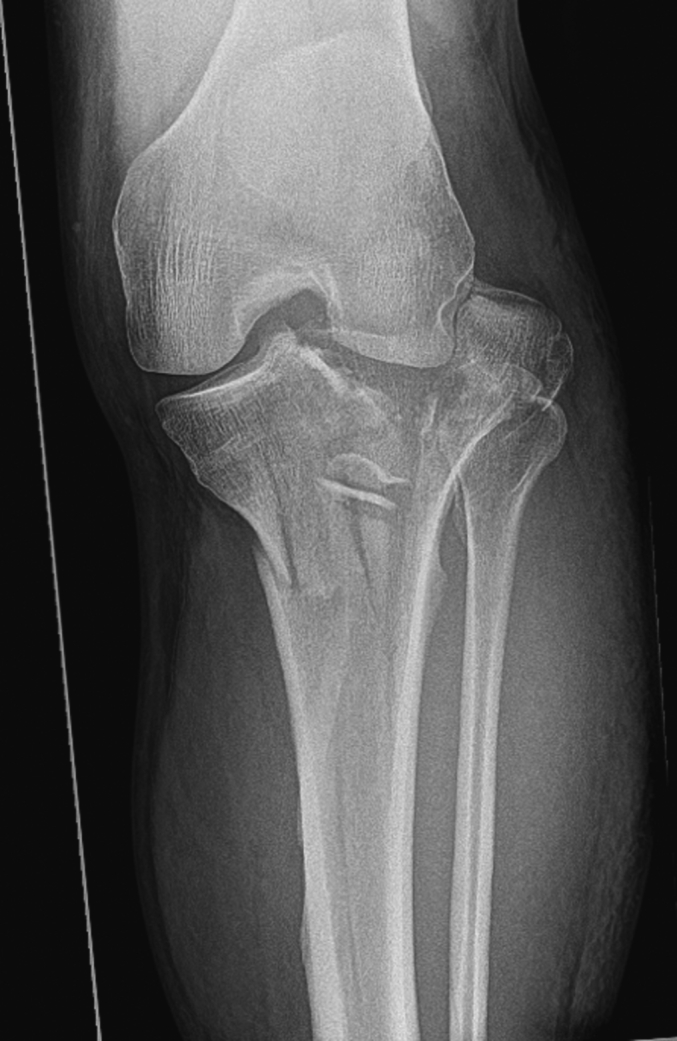
AP radiograph of a tibial plateau fracture-dislocation with partial loss of joint congruity.

In two patients there was complete loss of joint congruity due to a knee dislocation with anteromedial plateau fractures and fibular head avulsions (Fig. 3). In a further two cases there was partial loss of joint congruity with subluxation of the joint and displacement of the lateral femoral condyle into the fracture gap of the lateral plateau (Fig. 4).

These patients had an average time of follow up of 30 weeks, which was not significantly different to the length of follow up in the main group of patients (23 weeks). The neuropathy completely resolved spontaneously with time in all but two patients (82 %), who had had TPFs with concurrent knee dislocations. The average time for complete resolution of symptoms in those who did improve clinically was 7.55 ± 8.9 months, with a range of 1 to 24 months.

Three patients underwent nerve conduction studies, two of these were the patients with long-term issues, whilst the third one was the post-operative diagnosis patient, where it was carried out for diagnostic uncertainty. In all cases, the investigation confirmed the diagnosis of CPN injury. In the two patients with long-term issues, it confirmed there was a dense CPN lesion with recovery predicted to be unlikely, with one of them undergoing nerve grafting unsuccessfully. The third and last patient was the patient with the likely iatrogenic injury, where a minor neuropraxia was confirmed, and they experienced spontaneous resolution of all clinical symptoms within 6 months.

However, about half of these patients (n = 6, 56 %) had prolonged knee joint issues including ongoing pain, stiffness, and instability. This was a significantly higher proportion than the percentage of patients with similar long-term issues in the main group of plateau fractures (n = 228, 31 %, p = 0.01). Two of these patients (18 %) ended up needing further surgery to treat post-traumatic osteoarthritis in the form of a total knee replacement and an osteotomy, respectively. This was similar to the proportion of patients in the main group needing further surgery (n = 105, 14 %, p = 0.56).

## Discussion

In this study we documented a 1.47 % incidence of CPN palsy in association with tibial plateau fractures, most of which were associated with varus deformities. This incidence is slightly higher than a previous estimate in one previous study reported CPN palsy after knee injury [10].

Most fractures were classified as B-type TPFs (65 %). Specifically, the C3 fracture pattern was the most common type (35 %), with a significantly higher incidence than in the original TPF population (10.5 %). CPN injury was >2.5 times more likely to happen in bicondylar fractures (2.99 %) than in unicondylar fractures (1.14 %), and 4 times more likely to happen in C-type than B-type fractures.

In terms of the Luo classification, most fractures involved the medial column (either isolated or together with the lateral and posterior columns). This made fractures with medial column involvement significantly more likely to happen in this subset than in the main population of patients with TPFs, where lateral column injuries are more common [16]. In a similar way, the Schatzker IV medial split pattern, which encompasses all “medial only” injuries, was the most common type and accounted for 27 % of injuries. This was also shown when fracture side was assessed using the AO/OTA classification.

This essentially confirms the theory that medial tibial plateau fractures, despite being less common, are more likely to cause common peroneal nerve injuries. As described earlier, this is probably because the varus deformity is more likely to result in a mechanical stretch of the common peroneal nerve with a consequent injury. This was clearly seen when assessing CPN incidence in different types of fractures. It was 1.47 % across the whole group, 4.59 % in fractures involving the medial column of the tibial plateau, and 2.99 % in bicondylar fractures. In addition, the majority of fractures with associated CPN injury were found to have a varus deformity on initial assessment (91 %).

The prognosis for these patients is good, with over 81 % of our cohort recovering full function of their common peroneal nerve within 2 years and in an average time of 7.5 months. This is significantly better than CPN palsy in other knee injuries, in particular knee dislocation, where only half the patients fully recover their neurological function and only 21 % recover full neurological function [18].

Common peroneal nerve palsies are a rare complication of knee trauma, but can be looked out for in high-risk cases. However, because of this, there is no other study specifically looking at CPN injury in patients with TPFs, and it is only reported as case reports in the literature or as part of bigger projects looking at all kinds of knee trauma [6]. In particular, it is usually compared against patients with knee dislocations, where it has a higher incidence and a poorer prognosis, with most patients not recovering full common peroneal nerve function [18]. This is consistent with our findings, since the two patients with the poorest prognosis both had concurrent knee dislocations.

A major strength of this study is the fact that it was carried out in a large, urban Scottish population where all orthopaedic trauma treatment is provided from a single centre, with no other units in the region. This meant that all TPFs would be recorded in this centre's hospital database and easily found, yielding a very large patient cohort studied for an epidemiological study of tibial plateau fractures [12,19,20]. This study also includes an extensive analysis of all potentially relevant factors in the patients affected, including patient demographics and background, details of the injury, management, early and long-term complications. It also includes a detailed fracture assessment with three different classification systems, balancing the drawbacks that each one could have and limiting the errors that could have happened when classifying them [21,22].Another strength of our study is that there are no other published articles specifically studying a group of patients with concomitant tibial plateau fractures and CPN injuries.

However, some of the weaknesses in this study include the small sample size of the actual CPN patient cohort, and the fact that the study was carried out retrospectively. This could have led to some patients with CPN injuries being missed if it was not documented clearly on their notes, if they were lost to follow-up or no appropriate follow-up was arranged, or if it was dismissed by the clinician because it was considered a minor problem in cases of polytrauma. Nonetheless, all patients were followed up to fracture union at a single centre, which minimises the risk of missing a diagnosis of such a disabling injury. In addition, the absence of nerve conduction studies in most of the patients may have missed some minor persistent residual nerve compromise in some patients. However, on the basis of the clinical records none of those described as being recovered had any clinically significant residual disability so it seems likely any lingering damage was negligible from a functional standpoint.

## Conclusion

In conclusion, this study is the first epidemiological description of common peroneal nerve injuries associated with tibial plateau fractures, and the relationships to fracture pattern and outcome. The findings can be used to inform clinicians how to identify those fractures which a higher risk of CPN injury advise about prognosis. Patients can be advised that CPN palsy is rare in TPFs, usually happening in higher-energy injury patterns in male patients and should be looked for in medial and bicondylar TPFs, but the prognosis for recovery is very good.

Further research on this topic should be done prospectively, possibly combining these factors into a risk scoring tool to ensure identification and appropriate management of CPN injuries in all high-risk patients, as well as a follow-up monitoring of these CPN injuries to advise patients of the most likely course of improvement.

## Declaration of competing interest

The authors declare no conflict of competing interest.

The authors would like to acknowledge The University of Edinburgh for covering the publication costs as a supporting funding body through their endowment fund.
